# Metformin-associated lactic acidosis exacerbated by acute kidney injury in an overseas traveler

**DOI:** 10.1007/s13730-021-00665-z

**Published:** 2021-11-27

**Authors:** Ayano Hayashi, Takuya Ishimura, Hisashi Sugimoto, Hiroyuki Suzuki, Akihiro Hamasaki, Tatsuo Tsukamoto

**Affiliations:** 1 Department of Nephrology and Dialysis, Kitano Hospital, Tazuke Kofukai Medical Research Institute, 2-4-20 Ohgimachi, Kita-ku, Osaka, 530-8480 Japan; 2 Department of Nephrology, Graduate School of Medicine, Kyoto University, 54 Shogoin-Kawahara-cho, Sakyo-ku, Kyoto, 606-8507 Japan; 3 Kidney Disease and Transplant Center, Shonan Kamakura General Hospital, 1370-1 Okamoto, Kamakura-city, Kanagawa 247-8533 Japan; 4 Center for Diabetes and Endocrinology, Kitano Hospital, Tazuke Kofukai Medical Research Institute, 2-4-20 Ohgimachi, Kita-ku, Osaka, 530-8480 Japan

**Keywords:** Metformin, Lactic acidosis, Angiotensin-converting enzyme, Acute kidney injury, Case report

## Abstract

We report the case of metformin-associated lactic acidosis (MALA) exacerbated by acute kidney injury (AKI) in a 65-year-old Asian American woman who was an overseas traveler. She had vomiting and diarrhea before arriving in Osaka, Japan, from the Philippines. She suffered from worsening respiratory distress, consciousness loss and anuria the day after coming to Japan. When she arrived at our emergency room via ambulance, she appeared to be in a state shock. Arterial blood gas analysis revealed severe lactic acidosis (pH 6.681, PO_2_ 302 Torr under O_2_ supplementation, PCO_2_ 15 Torr, HCO_3_^−^1.7 mmol/L, and lactate 17.00 mmol/L). She also had renal failure (BUN 108 mg/dL and serum creatinine 8.68 mg/dL) with hyperkalemia (6.1 mEq/L). We collected medical information from family members, and found her prescription medicines including metformin, diuretics and angiotensin-converting enzyme inhibitor (ACEI). We diagnosed her with MALA due to an unintended overdose of metformin resulting from acute kidney injury that can be induced by ACEI and diuretics in the volume-depleted condition. We immediately started hemodialysis therapy. Although she had a temporary cardiopulmonary arrest at the beginning of the treatment, her physical status was gradually improved and the severe acidemia resolved. On hospital day 4, she had urine and no longer needed hemodialysis therapy. On day 14, she was discharged and returned to the United States without noticeable sequelae. This is a case report of an overseas traveler who was successfully rescued through the collection of accurate medical information and understanding of the pathological condition.

## Introduction

Metformin is a worldwide first-line agent for type 2 diabetes mellitus (DM). In the United States, the first-line use of metformin increased after 2005 according to Centricity Electronic Medical Records [1]. Metformin is a relatively safe drug for diabetic patients with normal renal function, but if patients have renal impairment, dose reduction or discontinuation is required. One of the severe adverse effects is metformin-associated lactic acidosis (MALA), which has an annual incidence of less than 10/100,000 patients [2]. MALA is thought to be less frequent in Japan. Since the MALA-associated mortality rate is 30 to 50%, rapid and appropriate treatment including blood purification is required [3].

Angiotensin-converting enzyme inhibitor (ACEI) and angiotensin receptor blocker (ARB) are representative drugs used to treat hypertensive DM patients who have micro- or macro-proteinuria [4]. The combination of ACEI or ARB with diuretics is a common treatment for diabetic kidney disease patients who tend to have fluid overload, however, this prescription can cause acute kidney injury (AKI) under volume depletion [5]. In particular, ACEI accumulates in the body when renal function declines. Since inhibition of the rein-angiotensin-aldosterone system in the volume-depleted condition leads to severe hypotension, ACEI may be an exacerbating factor for AKI.

Here, we report a case of MALA in an overseas traveler that was successfully rescued by rapid diagnosis and appropriate treatment. It is very important to collect medical information, especially in regard to therapeutic agents, in the case of overseas travelers with sudden illness. This case was instructive in the following three points: (1) the importance of collecting medical information, (2) the difference in metformin dose between Japan and the United States, and (3) the background conditions for the onset of MALA.

## Case report

A 65-year-old Asian American woman with type 2 diabetes mellitus (DM) visited Osaka, Japan via the Philippines for sightseeing. She had diarrhea, poor appetite, and vomiting the day before her arrival in Japan. She also had hypoglycemia because of her poor appetite, and took glucose several times at her hotel. She suffered from worsening respiratory distress, loss of consciousness, and anuria upon arrival in Japan. When she visited our emergency room via ambulance, she was in a shock state. We obtained her medical history of type 2 DM, diabetic retinopathy and nephropathy, and ischemic heart disease from her family. We were unable to determine the details of her daily use medications at this time. Her height was 151.5 cm and her estimated body weight was 50.0 kg. Her level of consciousness was at Glasgow Coma Scale E3V4M5. Her blood pressure was 67/42 mmHg and pulse rate was 156 beats per minute. Her respiratory rate was 29 breaths per minute. Although her body temperature was normal at 36.0 °C, her extremities were cold and cyanotic. Laboratory examination revealed severe renal failure: blood urea nitrogen (BUN) 108.7 mg/dL serum creatinine (Cr) 8.68 mg/dL and hyperkalemia (6.1 mEq/L). Arterial blood gas analysis indicated severe acidemia: pH 6.68, PO_2_ 302 Torr, PaCO_2_ 15 Torr, bicarbonate (HCO_3_^−^) 1.7 mmol/L, and lactate 17 mmol/L, indicating severe lactic acidosis (Table 1).

**Table 1:** Laboratory examination results on admission

Biochemistry		Complete blood count		Blood gas (O_2_ 10L/min mask)	
AST	51 U/L	WBC	13,000 /µL	pH	6.681
ALT	41 U/L	Neutro	81.0%	PaCO_2_	15 Torr
LDH	278 U/L	Lympho	9.7%	PaO_2_	302 Torr
ALP	162 U/L	RBC	319 × 10^4^/µL	HCO_3_^−^	1.7 mmol/L
γ-GTP	23 U/L	MCV	110.0 fL	AG	37.8 mmol/L
TP	6.5 g/dL	Hb	10.2 g/dL	Lactate	17.0 mmol/L
ALB	3.9 g/dL	Plt	30.8 × 10^4^/µL		
CK	166 U/L				
CK-MB	58 IU/L				
Glu	164 mg/dL	Coagulation test			
T-CHO	164 mg/dL	PT-INR	1.39		
BUN	108.7 mg/dL	APTT	33.6 s		
UA	13.8 mg/dL	D-dimer	3.6 μg/dL		
Cre	8.68 mg/dL				
Na	138 mEq/L				
K	6.1 mEq/L				
Cl	93 mEq/L				
Ca	9.5 mg/dL				
P	19.9 mg/dL				
CRP	0.8 mg/dL				

**Fig. 1:**
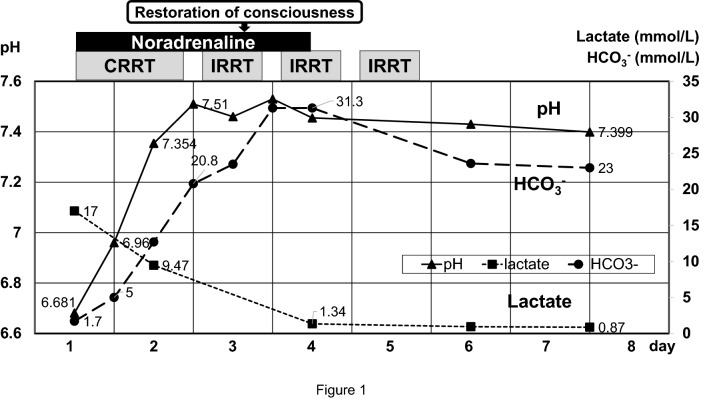
Clinical course. Changes in arterial blood pH, HCO_3_^−^, and lactate levels over time are plotted along the clinical course. The patient regained consciousness on hospital day 3 and her kidney function recovered on hospital day 6 following multidisciplinary treatment, including blood purification therapy

After examining her cardiac function by electrocardiogram and echocardiography, we started fluid resuscitation and catecholamine administration. We used 1500 mL saline together with bicarbonate for three hours until blood pressure rose above 100/mmHg to correct volume depletion and metabolic acidosis. Despite the rapid fluid administration, her blood pressure did not go up, so we started the intravenous administration of noradrenalin. She had a cardiopulmonary arrest (CPA) at the beginning of the treatment, but we were able to get her ROSC (return of spontaneous circulation) quickly by standard cardiopulmonary resuscitation. Simultaneously, we collected medical information from family members, and found that her daily medications including metformin (2000 mg/day), glimepiride (8 mg/day), hydrochlorothiazide (25 mg/day), lisinopril (unknown dosage), amlodipine (unknown dosage), aspirin (unknown dosage) and rosuvastatin (5 mg/day). We diagnosed her with MALA due to an unintended overdose of metformin resulting from AKI that can be induced by ACEI and diuretics under volume depletion. We decided to start continuous renal replacement therapy (CRRT) to correct her highly impaired acid–base balance in our intensive care unit. After the initiation of CRRT, the severe acidemia and lactate levels were resolved quickly. Her vital signs, including blood pressure and respiratory status, also gradually recovered. On hospital day 2, her lactate levels decreased to 9.47 mmol/L and pH levels returned to 7.354 and she regained consciousness. We switched CRRT to intermittent renal replacement therapy (IRRT). Noradrenalin administration was also tapered. We did not determine her dry weight during blood purification therapy. The fluid removal on each dialysis session was set by referring to chest X-rays, edema, and the amount of infusion volume. When she got out of bed before returning to the US, her body weight was 50.4 kg. We stopped hemodialysis therapy on hospital day 4 after normalization of her metabolic state (Fig. 1).

Her activities of daily livings were restored by rehabilitation without any neurological sequelae after CPA. Diarrhea, vomiting and other gastrointestinal symptoms that caused her sick day were spontaneously recovered without any special treatment. On hospital day 14, her serum creatinine levels decreased to 1.55 mg/dL. She returned to the US with her family. Her attending physician in Los Angeles, CA, subsequently informed us that her baseline creatinine level was 1.3 mg/dL before coming to Japan.

## Discussion

We report a case of MALA in an overseas traveler successfully rescued by the rapid collection of medical information and appropriate hemodialysis therapy. This case was very instructive because details related to medical information and underlying diseases are limited when overseas travelers suddenly become severely ill. This case provided three important lessons.

The first lesson related to the importance of obtaining the patient’s medical history. In this case, we were initially unable to determine the patient’s medical history because of her severe loss of consciousness. We were also unable to obtain sufficient information from her family. As such, we had to infer her disease status by examining the prescribed medications she had with her (the prescription dose was unknown). As she went into CPA in our emergency room, we did not have time to interview her family. We assumed that she might have developed shock with severe lactic acidosis as a result of excess metformin intake.

Although metformin poisoning can cause severe toxicity, but there is no specific antidote to reverse the toxic effects. One treatment option for MALA is sodium bicarbonate infusion. An intravenous administration of 1 to 2 mEq/kg BW sodium bicarbonate and a repeat administration at the same dosage after 30 to 60 min if the pH level remains below 7.1 is recommended [6]. Sodium bicarbonate did not extend patient life expectancy [6]. The other treatment option for MALA is hemodialysis therapy. The criteria for extracorporeal treatment (ECTR) were proposed by the Poisoning Workgroup [7]. When lactate level is > 20 mmol/L, pH is ≦ 7.0 and standard therapy (supportive measures, bicarbonate, etc.) fails, ECTR is recommended. On the other hand, when lactate level is 15 to 20 mmol/L, pH is 7.0 to 7.1, and there is impaired kidney function, shock, decreased level of consciousness, and liver failure, ECTR is suggested. ECTR discontinuation is indicated when the lactate level is < 3 mmol/L and pH is > 7.35. In our case, the pH alone met the recommended criteria for ECTR, and the other symptoms met the suggested criteria for ECTR. Given the cession criteria for ECTR, the lactate level was resolved to 1.9 mmol/L and the pH level was resolved to 7.53 on hospital day 2. We continued dialysis therapy until hospital day 5 because of her anuric condition (Fig. 1).

The prognosis of MALA can change depending on the modality of blood purification. Previously, 41 of 253 cases were reported to die and the mortality was 16.2% [8]. There were no differences in metformin dose, age, or modality of ECTR between the surviving and deceased groups, and the lactate levels and creatinine levels at the first arrival were higher in the deceased groups. This report indicated that lactate levels above 20 mmol/L may be associated with the mortality. In our case, we chose CRRT to treat severe lactic acidosis and shock with catecholamine support following the ROSC, which likely would have made it difficult to perform IRRT. Although her lactate level at the time first arrival was 17 mmol/L when she presented to the emergency room, this could have increased when we started CRRT.

Second, the regular and maximum dose of metformin in the US and England is much more than that in Japan (Table 2). In Japan, a daily dose of metformin is 750 to 1500 mg, and a maximum daily dose is 2250 mg. The use of metformin is contraindicated if eGFR is less than 30 mL/min/1.73m^2^ and it can be given carefully if eGFR is 30 to 45 mL/min/1.73m^2^ according to the “Recommendation for the proper use of metformin” of the Japan Diabetes Society [9]. Dose reduction is also required depending on renal functions. The maximum daily dose is 750 mg if eGFR is 30 to 45 mL/min/1.73m^2^, and it is 1500 mg if eGFR is 45 to 60 mL/min/1.73m^2^. On the other hand, metformin use is not recommended to start in renal impairment (eGFR 30 to 45 mL/min1.73m^2^) in the US, the maximal dose in the US and England is 1000 mg/day. Indeed, the efficacy of metformin use in patients with renal impairment has been reported. A retrospective cohort study of US veterans receiving care within the national Veterans Health Administration showed that in type 2 DM patients with renal impairment, metformin-alone therapy has a lower risk of major adverse cardiovascular events (MACEs) than sulfonylurea (SU) therapy; the use of metformin in encouraged in patients with mild to moderate renal impairment [10]. Another cohort study in the Geisinger Health System that considered the association between metformin use across all ranges of eGFR considering the aging changes and hospitalization due to lactic acidosis has suggested that metformin use was associated with acidosis only at eGFR less than 30 mL/min/1.73m^2^ [11]. In our case, the baseline renal function (eGFR) was between 30 and 45 mL/min/1.73m^2^. The maximum daily dose of metformin was 750 mg/day in our case in Japan, but she was actually prescribed at 2000 mg/day. Although the patient was American, she weighed 50 kg, and that dose of metformin would have been excessive. It should be noted that larger doses of metformin are prescribed outside of Japan. It is necessary to consider the possibility of overdose in cases of small body size, as in this case.

**Table 2:** Difference in daily maximal dose of metformin in a patient with impaired kidney function among Japan, the United States, and England

Kidney function	Japan	United States	England
60 ≤ eGFR < 90	2250	3000	3000
45 ≤ eGFR < 60	1500	2000	2000
30 ≤ eGFR < 45	750	1000/Not recommended to start	1000
< 30	Contraindicated	Contraindicated	Contraindicated

Third, the safety of metformin should be considered in a case with rapidly declining renal function. The patient had diarrhea, poor appetite, and vomiting the day before her arrival at Japan, leading to volume depletion. She took ACEI (lisinopril) and diuretics (hydrochlorothiazide) in addition to metformin. MALA is more likely to occur in patients who developed AKI by dehydration, vomiting or diarrhea, surgery, etc. [12]. Volume depletion can cause AKI and reduce metformin clearance, resulting in increased plasma metformin levels [13]. It is known that the combination of ACEI/ARB and non-steroidal anti-inflammatory drugs (NSAIDs) or diuretics is also likely to cause AKI [14]. Indeed, metformin, ACEI/ARB, and diuretics are commonly used in patients with diabetic nephropathy. Therefore, when prescribing a combination of these drugs, it is important to instruct patients not to take them when they are ill, especially when they are in a volume-depleted condition.

## Conclusion

Here, we report a case of MALA in an overseas traveler who was successfully rescued by rapid diagnosis and appropriate blood purification. When a foreigner with DM suddenly becomes ill, clinicians must investigate the difference in metformin dose between Japan and other countries, and the background conditions leading to the onset of MALA.

## Abbreviations

CRRT: Continuous renal replacement therapy
IRRT: Intermittent renal replacement therapy
